# Immunotherapy combined with chemotherapy without locoregional radiotherapy in *de novo* metastatic nasopharyngeal carcinoma: two case reports and a literature review

**DOI:** 10.3389/fimmu.2024.1467355

**Published:** 2025-01-09

**Authors:** Yingna Gao, Xiaoqiong Shi, Jianqiao He, Hui Yao, Guoning Yu, Lin Zhao, Yi Ma, Hongliang Zheng, Minhui Zhu, Caiyun Zhang

**Affiliations:** ^1^ Department of Otolaryngology, Changhai Hospital, Naval Medical University, Shanghai, China; ^2^ Department of Health Management Center, Shanghai Eastern Hepatobiliary Surgery Hospital, Shanghai, China; ^3^ The 909th Hospital, School of Medicine, Xiamen University, Zhangzhou, China

**Keywords:** immunotherapy, chemotherapy, *de novo* metastatic nasopharyngeal carcinoma (dmNPC), Epstein-Barr virus (EBV), locoregional radiotherapy

## Abstract

**Background:**

There is no consensus regarding the optimal regimen for *de novo* metastatic nasopharyngeal carcinoma (dmNPC). Locoregional intensity modulated radiotherapy (LRRT) following palliative chemotherapy (PCT) has been shown to prolong the overall survival (OS) and improve the progression-free survival (PFS) of patients with dmNPC, compared with PCT alone. However, patients with a high tumor burden do not benefit from additional LRRT, which inevitably results in toxicity. Recently, immunotherapy has made great progress in the treatment of recurrent or metastatic NPC (RM-NPC). Compared with PCT alone, programmed death-1(PD-1) inhibitors combined with PCT have shown a promising survival outcome and an acceptable safety profile. Therefore, this treatment strategy is recommended as a first-line therapy for RM-NPC. However, whether dmNPC can be treated with immunochemotherapy alone (without LRRT) remains controversial.

**Case presentation:**

We report two cases of dmNPC, both in middle-aged men who mainly presented with epistaxis and systemic pain. Radiological examination with positron emission tomography–computed tomography (PET-CT) and contrast-enhanced magnetic resonance imaging (MRI) showed NPC with multiple systemic lymph node metastases, multiple bone metastases, and liver metastases. Both patients were diagnosed with dmNPC and received pabolizumab in combination with six courses of platinum-based chemotherapy treatment. After complete remission (CR) was achieved, the patients were maintained on pabolizumab alone. No LRRT was used throughout the course of the disease. Pre- and post-treatment levels of plasma Epstein-Barr virus (EBV) DNA were measured, and radiological imaging was performed before and after treatment.

**Results:**

We achieved good efficacy in these two cases of dmNPC. Both patients exhibited survival benefits (PFS has reached 31 months since diagnosis), and no serious chemotherapy- or immune-related adverse reactions occurred. Treatment-related toxicity from radiotherapy was avoided. Levels of plasma EBV DNA decreased and remained below the minimum detection level consistently after four or five cycles of treatment, with no obvious symptoms of neck muscle fibrosis, throat mucosa dryness, ear congestion, or nasal congestion.

**Conclusion:**

Our findings suggested that chemotherapy combined with a PD-1 inhibitor without LRRT, followed by sequential immunotherapy as maintenance, can achieve good results in some dmNPC patients. Further validation of our results may be required in large, high-quality prospective clinical studies.

## Introduction

Nasopharyngeal carcinoma (NPC), a malignant tumor occurring in the nasopharynx, originates from the nasopharyngeal epithelium (1). It is closely associated with Epstein-Barr virus (EBV) infection and has significant geographic and ethnic differences, as well as a tendency to exhibit family aggregation (2, 3). NPC is endemic in southeast Asia and southern China, especially in Guangdong, Guangxi, Hunan, Fujian, and Jiangxi (4). According to the latest Global Cancer Statistics, more than 130,000 new NPC cases are diagnosed globally annually, with roughly 4%–10% presenting with *de novo* metastatic nasopharyngeal carcinoma (dmNPC) (5, 6). Bone is the most frequently observed organ affected by metastasis in mNPC patients (54%–67%); two other common sites include the lungs (15%–22%) and liver (29%–36%) (7). Distant metastasis has been the major cause of treatment failure and death in NPC patients, and currently, no effective treatment exists (5, 8). Unlike patients with early and locally advanced NPC, whose five-year overall survival (OS) reaches up to 85%, those with dmNPC have limited treatment options and inferior survival outcomes, with a median OS of about 12 months (2, 8, 9).

Currently, owing to the complex and highly heterogeneous nature of dmNPC, there is no consensus regarding the therapeutic strategy (9–11). Platinum-based palliative chemotherapy (PCT) is the cornerstone of treatment, and gemcitabine combined with cisplatin (GP) is considered the standard first-line treatment for recurrent or metastatic NPC (RM-NPC) (11). However, the clinical efficacy of PCT alone is not ideal, and the duration of its response and survival time remain limited, with the objective response rates of 55%-80 (3), and the 3-year OS rate of only 20-30% (10), while the OS is approximately 10-15 months (12–14). Studies have shown that the addition of radiotherapy in the primary tumor and lymph node region can significantly improve OS, with the 3-year OS rate increasing from 12.4% to 48.3% (3), while the 5-year median OS time increasing to 21–36 months (15). Nevertheless, not all dmNPC patients benefit from locoregional intensity modulated radiotherapy (LRRT). Sensitivity to chemotherapy is key in selecting patients for LRRT (11). Additionally, radiotherapy in the head and neck region may cause severe oral mucositis, which affects eating and may induce the progression of disease due to poor nutritional status. Therefore, new treatment models are urgently needed. Cancer-specific cytotoxicity immunity is thought to play an important role in preventing the development and progression of cancer. Over the past decade, novel therapies that modify immunity, such as immune checkpoint inhibitors (ICIs), have dramatically changed the standard treatments of various cancers (11). The combination of PCT and ICIs has been widely used in clinical practice and has become the standard first-line treatment for mNPC (13). Studies have also shown that the median PFS was 28 months of patients with dmNPC who received PCT combined with anti-PD-1followed by LRRT and concurrent anti-programmed death-1 (PD-1) (2). Nevertheless, there is a lack of evidence-based medical proof to support the use of LRRT for all dmNPC patients in the era of immunotherapy.

Studies have clarified that plasma EBV DNA levels are correlated to NPC, which can be used as a powerful and easily accessible therapeutic monitoring, and a prognostic prediction marker of NPC (2, 14, 15). Dynamic surveillance of EBV DNA can reflect the tumor load in real time (2). Elevated levels of plasma EBV-DNA, have been found to predict poor prognosis. Oppositely, plasma EBV-DNA with faster clearance rates suggests better treatment response and patient outcomes (1). In this study, pre- and post-treatment plasma EBV DNA levels of the two patients with dmNPC were detected.

In this study, two patients with dmNPC were treated with PCT plus the PD-1 inhibitor pabolizumab, without LRRT. Both achieved sustained complete remission, and EBV DNA levels were continuously below the lower detection limit. Their survival time exceeded 2 years, with high quality of life, and radiotherapy-related toxicity and side effects were avoided. Furthermore, the treatment was well tolerated, and no serious chemotherapy- or immune-related adverse reactions occurred.

## Case presentation

**Figure 1:**
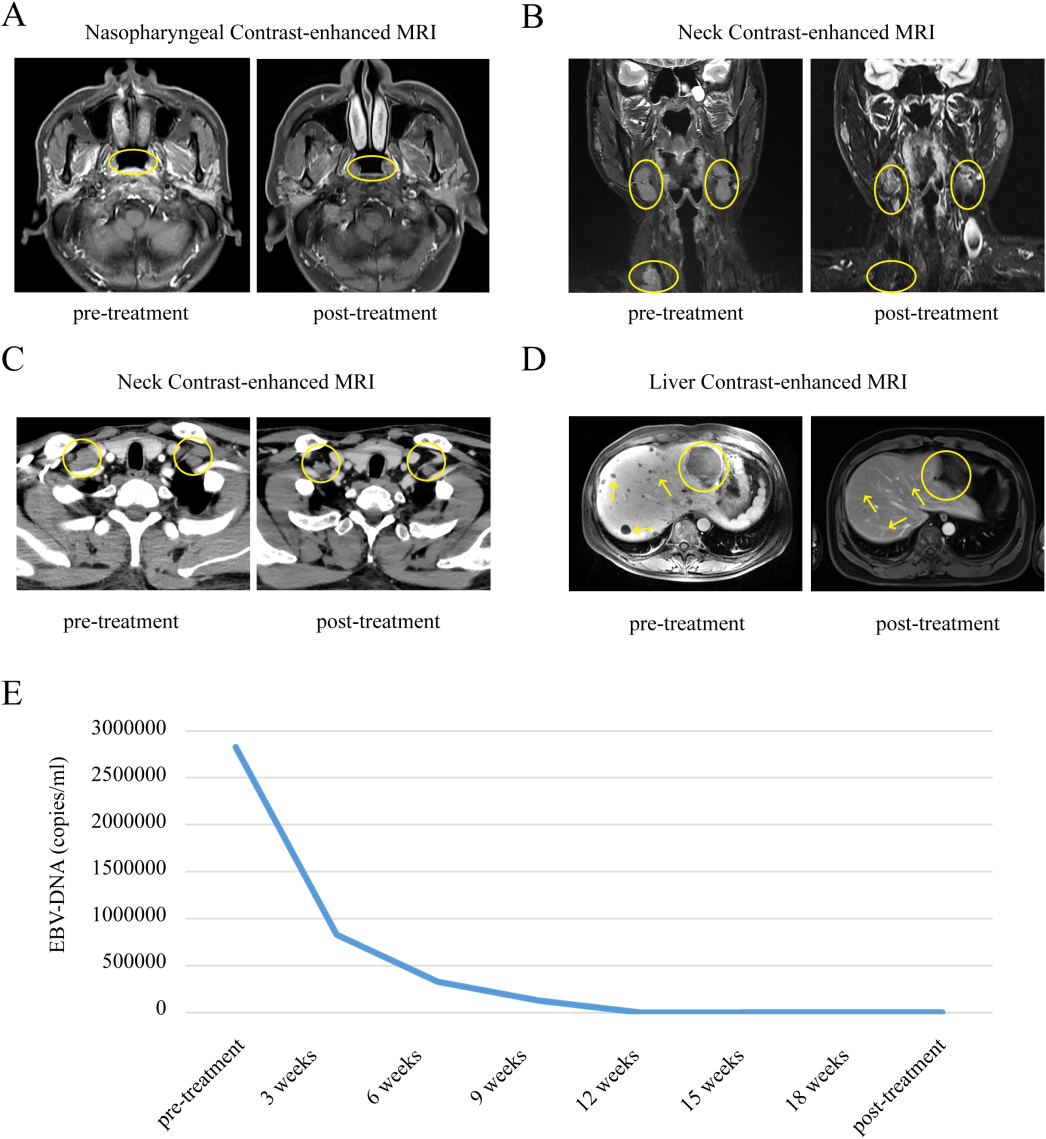
Comparison of changes in nasopharyngeal, neck and liver contrast-enhanced MRI, EBV-DNA before and after treatment in patient 1. **(A)** Comparison of nasopharyngeal contrast-enhanced MRI changes in patient 1 before and after treatment. **(B, C)** Comparison of neck contrast-enhanced MRI changes in patient 1 before and after treatment. **(D)** Comparison of liver contrast-enhanced MRI changes in patient 1 before and after treatment. **(E)** Longitudinal EBV-DNA titer before and after treatment of disease. The unit of EBV DNA levels was copies/mL. EBV, Epstein-Barr virus.

Case 1: In September 2021, a 48-year-old Chinese man with no family history of cancer presented with a one-month history of repeated epistaxis without obvious inducement, associated with pain all over the body, mainly in the left rib area. Electronic nasopharyngoscopy revealed that the surface of the top of the posterior nasopharynx was rough. Histopathology of a nasopharynx specimen showed nasopharyngeal poorly differentiated non-keratinizing squamous cell carcinoma. Positron emission tomography–computed tomography (PET-CT) showed incrassation in the posterior nasopharynx with high β-2-[18 F]-Fluoro-2-deoxy-D-glucose (FDG) uptake, systemic lymph node metastasis, multiple bone metastases, and multiple liver metastases (bile duct carcinoma of the left outer lobe of liver had to be ruled out). Contrast-enhanced magnetic resonance imaging (MRI) showed multiple liver metastases and metastatic tumors of the thoracic cone and adnexa. The patient was thus diagnosed with NPC stage T1N2M1 IVb. The pre-treatment level of plasma EBV DNA was 2.83×10^6^ copies/ml. Since diagnosis, he has received six rounds of PCT (paclitaxel protein-bound 230mg/m^2^ + cisplatin 75mg/m^2^) combined with immunotherapy (pabolizumab 200 mg) once every 3 weeks, followed by pabolizumab 200 mg every 3 weeks for maintenance. No LRRT was performed during the course of the disease. After 33 months of follow-up, the patient was in continuous complete remission with a stable condition. Recent nasopharyngeal contrast-enhanced MRI showed slight thickening of the right posterior nasopharynx with less uniform bone density on the slope. Liver contrast-enhanced MRI showed multiple liver metastases, which were significantly smaller than they were before treatment. The level of EBV DNA was consistently below the minimum detection limit (**Figure 1**).

**Figure 2:**
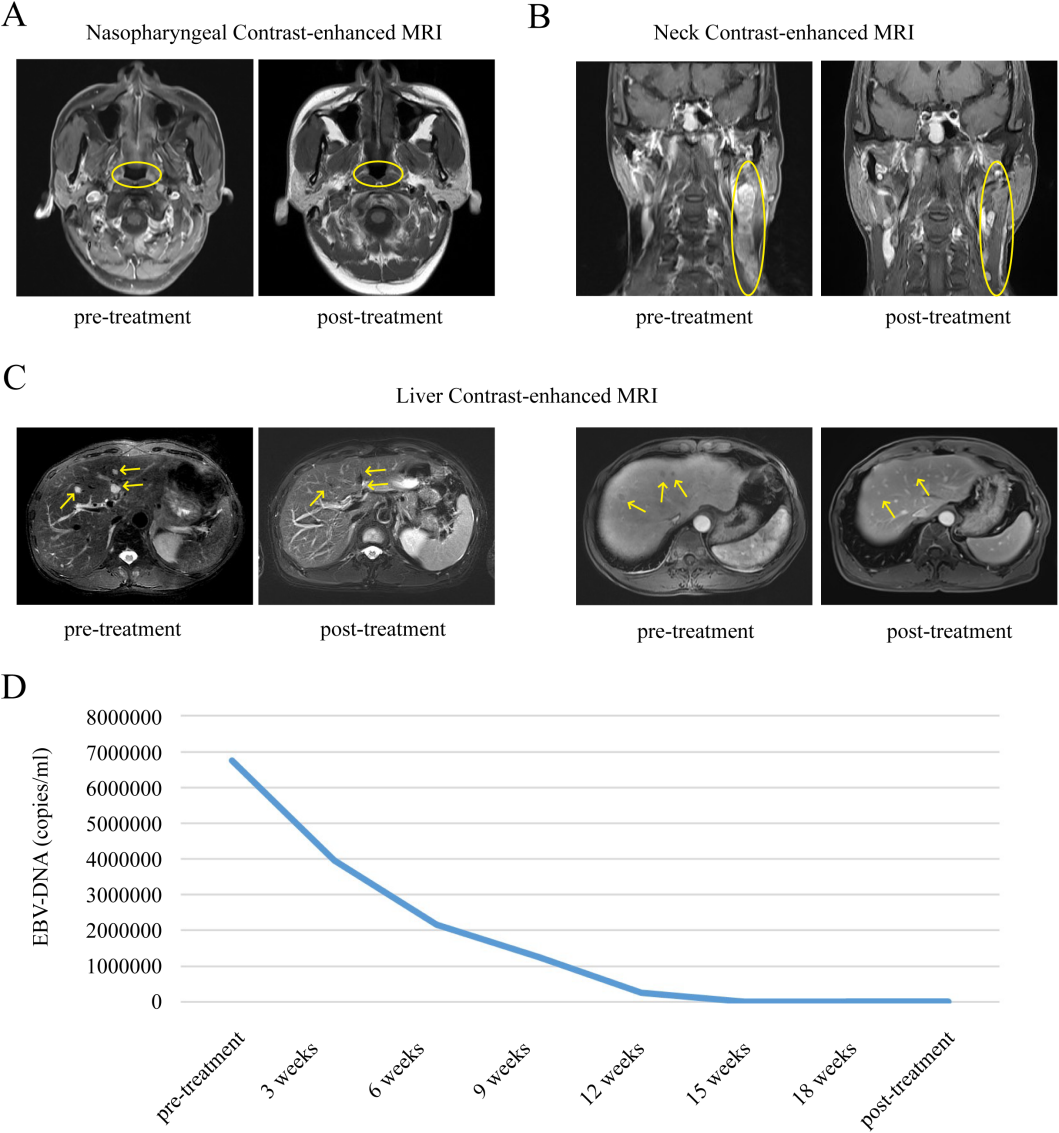
Comparison of changes in nasopharyngeal, neck and liver contrast-enhanced MRI, EBV-DNA before and after treatment in patient 2. **(A)** Comparison of nasopharyngeal contrast-enhanced MRI changes in patient 2 before and after treatment. **(B)** Comparison of neck contrast-enhanced MRI changes in patient 2 before and after treatment. **(C)** Comparison of liver contrast-enhanced MRI changes in patient 2 before and after treatment. **(D)** Longitudinal EBV-DNA titer before and after treatment of disease. The unit of EBV DNA levels was copies/mL. EBV, Epstein-Barr virus.

Case 2: In November 2021, a 49-year-old Chinese man presented with a three-month history of a lump in the neck region. Histopathology findings showed metastatic carcinoma of the neck. PET-CT showed multiple enlarged lymph nodes in the left parapharyngeal space, left II-V area, and left retroperitoneal abdominal aorta; multiple low-density lesions in the liver with high glucose metabolism; multiple lesions with high glucose metabolism in the bilateral scapula, the sternum, multiple ribs, multiple cones, the appendages, and the pelvic bones; and slightly thickened soft tissue in the left posterior nasopharynx, considered as primary NPC. Liver contrast-enhanced MRI showed multiple metastatic tumors in the liver, thoracolumbar spine, and pelvic bones, as well as slightly enlarged lymph nodes scattered retroperitoneally. Histopathology of a nasopharynx specimen showed nasopharyngeal poorly differentiated non-keratinizing squamous cell carcinoma. The patient was thus diagnosed with NPC stage T1N3M1 IVb. The pre-treatment level of plasma EBV DNA was 6.75×10^6^ copies/ml. Since diagnosis, the patient has received platinum-based PCT (gemcitabine 1000mg/m^2^ + cisplatin 75mg/m^2^) six times plus immunotherapy (pabolizumab 200 mg) once every 3 weeks, followed by pabolizumab 200 mg every 3 weeks for maintenance, without LRRT. After 31 months of follow-up, the patient was in continuous complete remission. Recent nasopharyngeal, neck, and liver contrast-enhanced MRI showed no significant abnormalities in the nasopharynx or liver, with slightly larger lymph nodes scattered in the left neck region. The level of EBV DNA was consistently below the minimum detection limit (**Figure 2**).

## Discussion

With limited treatment means and a poor prognosis, dmNPC remains a crucial clinical challenge, owing to the lack of a standard treatment regimen. Advances in modern modalities have enabled the development of individualized treatment plans (8). Several reports have demonstrated the value of LRRT in patients with dmNPC (2, 4–6, 10, 16–18). Some new intensive systemic therapies, such as the administration of targeted agents or immunotherapy, might help control distant metastatic lesions (8, 19). However, although epidermal growth factor receptor (EGFR) is overexpressed in NPC and is considered an important therapeutic target, retrospective studies showed that the therapeutic effect of the anti-EGFR drugs cetuximab (CTX) or nituzumab (NTZ) in combination with PCT is similar to that of PCT alone. This treatment strategy has not been shown to improve the survival rate of dmNPC patients, and CTX/NTZ + PCT may aggravate acute mucous inflammation and skin reactions. Therefore, anti-EGFR drugs for dmNPC patients should be administered with caution (17, 20, 21).

Over the past few years, immunotherapy with PD-1 inhibitors has improved the treatment of RM-NPC. Clinical trials have shown that the combination of ICIs and chemotherapy might have a synergistic effect that shows better integral control and provides a greater survival benefit than chemotherapy alone, with controlled toxicity and acceptable safety (13, 22). Therefore, PD-1 inhibitors combined with PCT have gradually become a new standard first-line treatment for dmNPC and have been widely applied in clinical practice. However, for dmNPC patients, there remains a lack of large-scale medical evidence supporting the use of PD-1 inhibitors combined with PCT, either alone or with concurrent sequential LRRT.

Some current retrospective studies and small sample prospective studies have found that sequential LRRT following chemoimmunotherapy may be more effective than chemoimmunotherapy alone in improving the overall prognosis of dmNPC (2, 8, 10, 15, 23, 24). However, it is important to note that not all patients with dmNPC will benefit from additional LRRT; only in the low-risk group (undetectable EBV DNA and satisfactory tumor response post PCT) did patients who received LRRT after PCT have significantly better OS than those who did not receive LRRT after PCT. This might be because patients in the low-risk group were sensitive to PCT, and distant metastases were better controlled, making local control important for long-term survival. However, for patients in the high-risk group (detectable EBV DNA and/or unsatisfactory tumor response post PCT), additional LRRT might not be sufficient to control distal lesions post PCT (5, 8, 17). Therefore, in these patients, not only was there an absence of significant survival benefits post-PCT LRRT, but also there was the presence of increased irreversible toxicity and side effects, such as severe oral mucositis, neck muscle fibrosis, hearing loss, and brain damage (5, 7). Therefore, whether all patients with dmNPC need LRRT is currently controversial, and it is unclear who should be treated with LRRT in the era of immunotherapy. Whether LRRT is required even in patients who achieve complete remission after chemoimmunotherapy remains to be determined.

We reported two patients with dmNPC who were treated with chemoimmunotherapy and sequential immunotherapy with single drug maintenance without LRRT. Both patients gained survival benefits with an unaffected quality of life, and toxicity and side effects from radiotherapy were avoided, suggesting that chemotherapy combined with anti–PD-1 monoclonal antibody without radiotherapy can have a very good effect in some patients with dmNPC. Also, it is crucial to accurately screen patients with dmNPC to determine whether follow-up LRRT is warranted. Our results provide reference values for the formulation of a standard diagnosis and treatment protocol for dmNPC. Further validation of our results may be required in large high-quality prospective clinical studies.

Chen et al. reported that dmNPC with liver involvement, regardless of the presence of metastatic lesions, had the worst survival outcomes and that affected patients could not benefit from additional LRRT in terms of substantial OS improvement (1, 5, 19, 21). In our study, both patients with dmNPC exhibited hepatic metastasis before treatment and were treated with PCT + PD-1 without additional LRRT. They achieved a similar prognosis and ideal curative effect compared with patients who received PCT + ICI + LRRT. Another reason for not adding LRRT was the T1 staging of the primary lesion in each case; as the primary tumor load was not large, complete remission could be quickly achieved after PCT + ICI, and the response of the cervical lymph nodes was also favorable. In addition, in both cases, the lymph node stage was N2 or N3, and the affected lymph nodes were located below the plane of the lower margin of the cricoid cartilage. In one case, supraclavicular and superior mediastinal lymph nodes also exhibited metastasis. Owing to the location and wide scope of the lymph node metastasis, it may have been relatively difficult to control with radiotherapy.

Therefore, when deciding whether to administer LRRT, the T stage of the primary lesion should be considered, in addition to considering factors including the reaction to PCT + ICI (usually tumor regression after six cycles), EBV DNA level, tumor burden, site and number of metastases, and liver involvement. Patients with an early-stage primary lesion and a good response to PCT + ICI can be treated without LRRT. These findings provide an important basis for the formulation of personalized treatment strategies for dmNPC.

## Conclusion

Altogether, the benefits of chemoimmunotherapy and sequential immunotherapy with single drug maintenance without LRRT appeared to outweigh those of treatment combined with radiotherapy in some patients with dmNPC, especially in those with an early-stage primary lesion and a good response to PCT + ICI. Further large-scale research is warranted to confirm these findings.

## Data Availability

The raw data supporting the conclusions of this article will be made available by the authors, without undue reservation.
